# Salivary Gland Sarcoidosis: Systematic Review of Case Reports and Case Series

**DOI:** 10.3390/jcm14217539

**Published:** 2025-10-24

**Authors:** Nadin Abouseif, Mohamed Jaber, Reem B. Abdelsayed

**Affiliations:** 1 Department of Clinical Sciences, College of Dentistry, Ajman University, Ajman P.O. Box 346, United Arab Emirates; reem.reem@outlook.com; 2 Center of Medical and Bio Allied Health Sciences Research, Ajman University, Ajman P.O. Box 346, United Arab Emirates

**Keywords:** oral sarcoidosis, salivary glands, parotid gland, salivary gland sarcoidosis, major salivary glands, minor salivary glands

## Abstract

**Background**: Salivary gland sarcoidosis is a rare manifestation of systemic sarcoidosis that poses a challenge in terms of its diagnosis due to its similarities to disorders such as Sjögren’s syndrome, other granulomatous diseases, and infections. **Objective**: To systematically review reported cases of salivary gland sarcoidosis and summarize clinical presentation, diagnostic methods, treatments, and outcomes. **Methods**: We conducted a systematic search of PubMed, Scopus, Embase, ScienceDirect, and Medline for case reports and case series published up to April 2025. This review was registered with PROSPERO (CRD42024629263) and was conducted following PRISMA guidelines. Variables assessed included age, sex, presenting symptoms, location, duration of symptoms, treatment approaches, and outcomes. Study quality assessment was assessed using The Joanna Briggs Institute (JBI) Critical Appraisal tools. **Results**: A total of 28 articles involving 39 participants met the inclusion criteria, with a mean age of 42.7 years. Salivary gland sarcoidosis predominantly affected female patients (66.7%). The parotid gland was the most frequently involved site (82.1%). Common presenting features included glandular swelling that is usually painless, xerostomia, and facial palsy. Sarcoidosis was newly diagnosed in 82.1% of cases, primarily through histopathological examination revealing non-caseating granulomas. Systemic corticosteroids were the most common treatment. Outcomes were favorable in nearly all cases, with complete resolution post treatment or spontaneous remission without treatment. **Conclusions**: Salivary gland sarcoidosis predominantly affects middle-aged women, typically presenting as a painless parotid swelling and often serving as the initial sign of systemic disease. Diagnosis requires histopathological confirmation via biopsy, as serum ACE levels are insufficient alone. The prognosis is excellent, with most patients responding favorably to corticosteroids or even experiencing spontaneous resolution. This condition must be considered in differential diagnoses for persistent salivary gland swellings to ensure accurate diagnosis and prevent unnecessary interventions.

## 1. Introduction

Sarcoidosis is a complex, multisystem inflammatory disorder characterized by the formation of non-caseating granulomas within affected tissues. Despite decades of research, the precise etiology remains unknown. Current evidence suggests that sarcoidosis arises from a dysregulated immune response to unidentified antigens, influenced by a combination of genetic susceptibility and environmental exposures [1].

The epidemiology of sarcoidosis reveals geographic and demographic variability. Incidence rates are highest in Scandinavian countries (11–23 cases per 100,000 annually) and lowest in parts of Asia (0.5–1 case per 100,000) [2,3,4,5,6]. Gender and age also influence disease patterns, with peak incidence observed in men between 30 and 50 years and in women between 50 and 60 years, potentially reflecting the interplay of hormonal, genetic, and environmental factors [3]. Sarcoidosis often remains asymptomatic and is incidentally detected during routine examinations, and when symptoms do appear, they are diverse and organ specific [7,8,9,10].

The oral presentation of sarcoidosis is infrequent and typically asymptomatic, occasionally causing discomfort, pain, or impair function such as speech, swallowing, or chewing [11,12,13]. Salivary gland involvement represents a rare manifestation, reported in fewer than 6% of sarcoidosis cases [7]. The diagnosis of salivary gland sarcoidosis is particularly challenging due to its nonspecific presentation, which includes painless glandular swelling and xerostomia. Clinical presentation often mimics other autoimmune or inflammatory conditions, such as primary Sjögren, because patients may experience xerostomia, dry eyes, and glandular swelling [12,14]. Unlike Sjögren’s syndrome, which typically shows characteristic sonographic changes in 50–60% of cases, sarcoid-related glandular disease is usually self-limiting and lacks specific ultrasonographic features, complicating differential diagnosis [11,14]. Definitive confirmation requires a tissue biopsy showing non-caseating granulomas, typically obtained from the affected gland through a minimally invasive approach. Treatment is largely empirical and depends on disease severity and systemic involvement. Systemic corticosteroids remain the mainstay of therapy, although spontaneous remission can occur in mild cases. Overall, the prognosis is favorable, with most patients achieving complete recovery [9,10].

While systemic and pulmonary manifestations of sarcoidosis have been widely researched, salivary gland sarcoidosis remains underrepresented in the literature. Therefore, this systematic review aims to analyze published case reports to better understand the epidemiology, clinical presentation, diagnostic methods, treatment strategies, and outcomes of salivary gland sarcoidosis. By integrating data from existing literature, this review seeks to assist clinicians in improving diagnostic accuracy and informed patient management.

## 2. Materials and Methods

### 2.1. Protocol and Registration

This systematic review is registered in the PROSPERO international prospective database of systematic reviews in health and social care under the registration identification number [ID: CRD42024629263]. The study adheres to the PRISMA (Preferred Reporting Items for Systematic Reviews and Meta-Analyses) guidelines, ensuring comprehensive and transparent reporting [15].

### 2.2. Search Strategy and Information Sources

A comprehensive electronic literature search was carried out across five major databases: PubMed, Scopus, Embase, ScienceDirect, and Medline to identify relevant studies and reduce chance of missed studies. The search utilized targeted phrases such (‘salivary gland sarcoidosis’) OR (‘parotid sarcoidosis’) OR (‘submandibular sarcoidosis’) OR (‘minor salivary gland sarcoidosis’) OR (‘major salivary glands sarcoidosis’) as keywords in the Advanced Search Builder. Filters Applied: Language: English, Article types: Case reports, case series and population: Human, with no time limitations. The final search performed was in April 2025. Studies were chosen based on predetermined inclusion and exclusion criteria set by the authors.

Records were reviewed by title and abstract by two independent reviewers (N.A. and R.A.). The selected articles were read in full to assess their eligibility. A list of excluded studies was maintained and updated periodically to prevent selection bias between the two reviewers. When the reviewers had differing views, they resolved the issue through discussion, and a third senior reviewer (M.J.) was consulted if consensus could not be reached.

### 2.3. Eligibility

The studies included in this review met all the predefined criteria specified by the PECOS framework (“Population,” “Exposure,” “Comparison,” “Outcomes,” and “Study design”) (Table 1).

### 2.4. Data Extraction

After reviewing the titles and abstracts of the search results, selected articles were read in full to assess their eligibility. Prior to synthesis, the extracted data were standardized to ensure consistency across the included case reports and case series. Data extracted included author(s) and year of publication, sample data: age and gender, location, initial symptoms, time to presentation, diagnosis of sarcoidosis (new/pre-existing), treatment, and follow-up and outcome (Table 2). Outcomes were harmonized into standardized categories (resolved, recurred, spontaneous remission, or unclear). When the reviewers had differing views, they resolved the issue by reaching a mutual agreement. In instances where a consensus was not readily reached between the two review authors, a third review author (M.J.) served as an impartial arbitrator to resolve any discrepancies. No assumptions were made about unreported diagnostic methods, treatment regimens, or long-term outcomes.

### 2.5. Study Quality Assessment

To assess the risk of bias, two review authors (R.A. and N.A.) independently conducted quality assessments for each of the included studies. The critical appraisal of case reports was assessed according to The Joanna Briggs Institute (JBI) Critical Appraisal tools issued by the Faculty of Health and Medical Sciences at the University of Adelaide, South Australia [41]. The checklist included whether demographic details, clinical presentation, diagnostic procedures, treatment, and outcomes were fully described. Discrepancies were resolved through consultation with a third review author (M.J.) (Table 3).

## 3. Results

### 3.1. Literature Search

**Figure 1:**
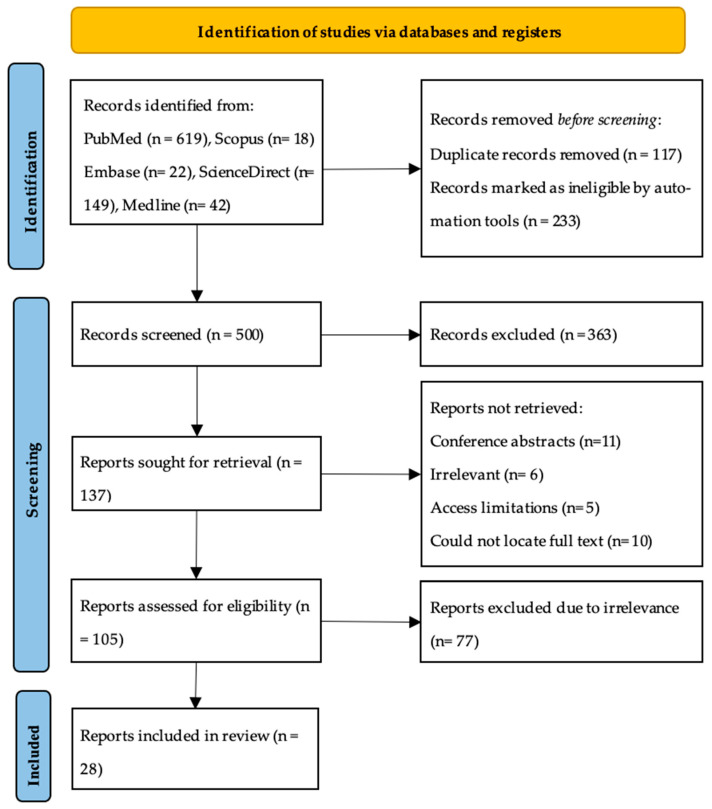
PRISMA flow diagram.

The databases’ search resulted in a total of 850 articles. After filtering for articles published of sarcoidosis in humans in English, and removing duplicates manually, 500 articles remained to be screened. After reviewing the title and abstracts, 363 were excluded. A list of excluded studies was maintained and updated periodically to prevent selection bias between two independent reviewers. The full texts of the remaining 137 articles were retrieved for assessment of eligibility. Of those 137 articles, 32 were not retrieved due to some being conference abstracts only (*n* = 11), a few were found to be irrelevant to the topic (*n* = 6), access limitations (*n* = 5), and some were older studies for which we could not locate the full text (*n* = 10). Leaving 105 articles to be further scrutinized according to our inclusion and exclusion criteria and to be critically appraised. Finally, a total of 28 articles were included in this systematic review following the inclusion criteria (Figure 1).

### 3.2. Study Characteristics

Twenty-eight articles that presented 39 patients met the inclusion criteria (Table 2). The 28 articles were case reports and case series published Between 1969 And 2024. Of the 28 articles, 24 were case reports, presenting 1–2 cases per article, while 4 were case series. The sample size was 39 patients diagnosed with salivary gland sarcoidosis, with an age range of 5–72 years and mean of 42.7 years. Females were more affected than male, accounting for 66.7% of our sample.

Diagnosis of salivary gland involvement was established through biopsy of the affected gland and histopathological confirmation. A detailed analysis of these 32 parotid cases revealed that swelling was most frequently bilateral, occurring in 20 cases (62.5%), compared to 12 cases (37.5%) that were unilateral. The swelling was predominantly described as painless (17/32, 53.1%) rather than painful (6/32, 18.8%), with pain status not specified in the remaining cases. Other involved sites included the submandibular gland (5.1%), minor salivary glands (2.6%), and multiple glands simultaneously (10.3%). In some cases, the swelling may be accompanied by xerostomia (33.3%), facial palsy (10.3%), dry eyes, and alteration in taste. Systemic symptoms associated included generalized fatigue (15.4%), loss of appetite and weight, and fever.

In the majority of cases (82.1%), the diagnosis of sarcoidosis was established following the presentation, while the remaining patients had a previously confirmed diagnosis. The duration from symptom onset to definitive diagnosis showed wide variability among reported cases, ranging from 1 week to 12 years. The median time to diagnosis was 3 months (interquartile range: 1–9 months) among 35 cases with available data, indicating that while most cases were identified within the first year, a subset experienced considerable diagnostic delays.

Treatment of choice was corticosteroids in 69% of cases; in very few cases (12.8%), surgical intervention was the chosen modality of treatment. In other cases, no treatment was undertaken, and a combination of surgery and corticosteroids was also the treatment choice in 2 cases (5.1%). Most of the cases showed favorable outcomes, with most resolving (79.5%) with treatment, and in a few instances spontaneous remission occurred (10.3%) where no treatment was provided. And only 1 case of recurrence occurred.

### 3.3. Study Quality Assessment

Included studies were assessed using The Joanna Briggs Institute Critical Appraisal tools for JBI systematic reviews [41]. The tool focuses on sufficient demographics, history, presentation, diagnosis, and proper intervention. Studies scoring 7–8 were deemed as having a “high quality,” scoring 4–6 was “moderate quality,” and scores of‚ <3 were “poor quality”. Of the 28 articles, 11 scored high quality and 17 scored moderate. Moderate scores were due to a lack of data on some criteria (Table 3).

## 4. Discussion

Salivary gland sarcoidosis represents a rare but clinically significant manifestation of systemic sarcoidosis. Histopathologically characterized by non-caseating granulomas, it may involve major salivary glands like parotid, submandibular, and sublingual glands. The findings reveal that although salivary gland involvement in sarcoidosis is rare, it presents with distinctive clinical features and demonstrates a variable response to treatment. Despite its rarity, salivary gland involvement may represent the initial or sole manifestation of sarcoidosis, underscoring the need for heightened clinical awareness.

Most patients were diagnosed at an average (SD) age of 42.7 (16.4) years, with most of them diagnosed during the fourth decade of their lives, with a market female predominance, consistent with previous literature [1,42]. Our analysis confirms that the parotid gland is the epicenter of salivary gland sarcoidosis, involved in over 80% of cases. The high prevalence of bilateral parotid enlargement (62.5% of parotid cases) is a particularly distinctive clinical feature. This bilateral, often painless swelling should raise immediate clinical suspicion for a systemic inflammatory or granulomatous process, such as sarcoidosis, as it is less commonly a feature of benign neoplasms, which are typically unilateral [43]. The predilection for the parotid gland may be related to its high lymphatic content and its role as part of the lymphoepithelial system, making it a potential site for the deposition of unidentified antigens that trigger the granulomatous response [1,44]. When a patient presents with bilateral parotid swelling, the differential diagnosis must be systematically evaluated to distinguish sarcoidosis from other causes such as Sjögren’s syndrome, IgG4-related disease, chronic sialadenitis, and metabolic disorders like diabetes mellitus. A smaller subset of cases involved the submandibular, and minor salivary glands, though these occurrences remain notably rare in published literature. Additionally, in some instances sarcoidosis did not just affect one gland but involved a number of glands at once [13,20,28,40].

The hallmark clinical presentation was swelling of the glandular tissue (in over 80% of the cases), which is frequently painless, but in a few cases, pain was noted although it was atypical [11,34]. This pain may possibly be attributed to localized restricted inflammation, nerve involvement (facial nerve for instance), or secondary infection [11]. This may be accompanied by xerostomia, which may be due to the replacement of salivary gland tissue with non-caseating granulomas, leading to decrease in saliva production hence dry mouth. Systemic manifestations including fatigue, low-grade fever, and weight loss were noted in a minority of cases (n = 5), while facial palsy occurred in 4 cases, which may be attributed to granulomatous infiltration or compression of the facial nerve.

Diagnosis of salivary sarcoidosis remains complex due to symptom overlap with conditions like Sjögren’s syndrome and benign salivary gland tumors. In most included reports, the diagnosis was confirmed through histopathological analysis demonstrating non-caseating granulomas within glandular tissue, which remains the gold standard [10,44]. These biopsies were obtained through surgical excision, incisional biopsies, or FNAC. The role of imaging was less frequently detailed but may support clinical suspicion in bilateral or symmetrical glandular swelling [44]. The diagnosis was often newly established (82.1%) in the context of the salivary gland presentation rather than a recurrence or known history of systemic sarcoidosis, indicating that salivary gland presentation association may be the first manifestation of systematic sarcoidosis. The non-caseating granulomas are composed predominantly of epithelioid histiocytes, often accompanied by multinucleated giant cells, and surrounded by a sparse rim of lymphocytes [45,46,47,48]. Importantly, these granulomas lack central necrosis, distinguishing them from infectious granulomatous diseases such as tuberculosis or fungal infections [49,50]. Elevated serum angiotensin-converting enzyme (ACE) levels are often used as a supportive biomarker in the diagnosis of sarcoidosis [50]. In this review, ACE levels were reported in 21 of the 39 cases, of which 15 demonstrated elevated values (71.4%), 5 were within normal limits, and one showed a decreased level. This rate of elevation is consistent with the known suboptimal sensitivity of this test, which literature suggests ranges from approximately 41% to 78%, depending on the population and disease activity [50,51]. While elevated ACE levels may reinforce clinical suspicion, particularly in cases with multisystem involvement, their sensitivity and specificity remain limited [51]. Normal ACE levels, as seen in several cases in this review, do not exclude sarcoidosis and were often present in patients with isolated salivary gland involvement or early-stage disease. Therefore, a normal serum ACE level should not deter a clinician from pursuing a diagnostic biopsy in the presence of suggestive clinical findings.

The clinical presentation of salivary gland sarcoidosis, particularly when accompanied by xerostomia and glandular swelling, creates a significant diagnostic overlap with other conditions, primarily Sjögren’s syndrome (SS) and IgG4-related disease (IgG4-RD). Accurate differentiation is critical as the management and long-term prognosis differ substantially. As summarized in Table 4, key distinguishing factors include serological markers, histopathological findings, and characteristic extraglandular manifestations. SS is typically seropositive for anti-SSA/Ro and anti-SSB/La antibodies and demonstrates focal lymphocytic sialadenitis on histology [52]. IgG4-RD is characterized by elevated serum IgG4 levels and histopathological findings of storiform fibrosis, obliterative phlebitis, and a dense IgG4+ plasma cell infiltrate [53]. In contrast, sarcoidosis is defined by non-caseating granulomas [45,46,47], lacks specific autoantibodies, and serum ACE may be elevated but lacks sensitivity [50,51]. Heerfordt’s syndrome (uveitis, parotitis, facial palsy, and fever) is a classic, though rare, extraglandular presentation of sarcoidosis [32,34], whereas SS is more commonly associated with arthralgia and Raynaud’s phenomenon [52]. A comprehensive evaluation incorporating these features is essential to avoid misdiagnosis and guide appropriate therapy.

Treatment most commonly included only non-surgical (steroids) treatment (82.1%), and these patients typically demonstrated favorable outcomes, with resolution or significant reduction in glandular swelling and systemic symptoms. This confirms the well-established role of corticosteroids in sarcoidosis management [7,10]. A subset of patients underwent surgical intervention, primarily when initial suspicion was directed toward neoplastic disease. In these cases, excision was not only diagnostic but also therapeutic. In other cases, no treatment was performed, and spontaneous remission of the lesion occurred, corroborating that sarcoidosis can follow a self-limiting course [1]. Combination approaches involving both surgical and medical therapies were also employed in a few instances [16,22].

A key clinical insight from this review is the validation of a ‘watchful waiting’ or observational approach in a select subset of patients. The spontaneous remission observed in 12.8% of our cohort, all of whom had isolated salivary gland disease without severe functional impairment or threatening complications, aligns with the known self-limiting nature of sarcoidosis in many forms [1,54]. This suggests that for patients with mild, non-progressive, and asymptomatic glandular enlargement, an initial period of observation is a reasonable and evidence-supported strategy. This approach avoids the potential side effects of corticosteroid therapy, such as hyperglycemia, weight gain, and osteoporosis, which are not insignificant [10]. The decision to initiate treatment should therefore be guided by the presence of symptoms (e.g., pain, significant xerostomia), cosmetic concern, progressive enlargement, or the development of systemic or organ-threatening manifestations (e.g., uveitis, facial palsy). Future studies should aim to identify predictive factors that can reliably distinguish patients who will undergo spontaneous remission from those who will progress, thereby further refining management guidelines.

Analysis of outcomes based on treatment type reveals a uniformly favorable prognosis for salivary gland sarcoidosis, irrespective of the management strategy employed. The majority of patients (27/39, 69.2%) were treated with corticosteroids, with 79.5% (31/39) of all cases achieving complete resolution. Within the corticosteroid group, the response was excellent, leading to resolution in nearly all treated cases. This confirms the well-established role of corticosteroids as the first-line therapy for symptomatic disease [7,10]. Notably, a significant subset of patients (5/39, 12.8%) received no treatment and experienced spontaneous remission. Surgical intervention, employed in 12.8% (5/39) of cases, was primarily diagnostic, often performed under the initial suspicion of a neoplasm; however, it proved curative in these isolated gland presentations. The combination of surgery and steroids was used in two cases (5.1%) with successful outcomes. This data underscores that while corticosteroids are highly effective, the natural history of salivary gland sarcoidosis can be self-limiting, and invasive procedures are often unnecessary once the diagnosis is confirmed.

Based on the findings of this review, we propose a practical diagnostic workflow for evaluating suspected salivary gland sarcoidosis. For a patient presenting with persistent, predominantly painless salivary gland swelling (especially bilateral parotid involvement), the initial assessment should include a detailed history for systemic symptoms (e.g., fever, fatigue, visual changes, respiratory complaints) and a physical examination. Initial serological testing should aim primarily at exclusion, targeting SS (anti-SSA/SSB) [52] and, if clinically suggestive, IgG4-RD (serum IgG4 levels) [53]. Serum ACE can be ordered but should not be relied upon for rule-out, given its limited sensitivity [52]. A chest X-ray or CT scan is crucial to identify concomitant hilar lymphadenopathy or pulmonary infiltrates, which strongly support the diagnosis of sarcoidosis [1,10,44]. However, the definitive diagnostic step remains a tissue biopsy of the affected salivary gland (or concomitant suspicious lymph nodes) to demonstrate non-caseating granulomas [10,45,46,47]. This approach ensures accurate diagnosis, facilitates appropriate systemic staging, and prevents unnecessary interventions for benign or self-limiting conditions.

This review highlights the importance of considering sarcoidosis in the differential diagnosis of painless, chronic salivary gland swellings, particularly in patients with associated constitutional or ocular symptoms. Histological evaluation should be pursued early, especially when imaging or serological testing is inconclusive. Multidisciplinary collaboration with oral medicine, rheumatology, and ophthalmology is essential for accurate diagnosis and systemic evaluation. Future multicenter registries and prospective studies are needed to better define the true prevalence and optimal management of this rare presentation.

## 5. Limitations

Several limitations must be acknowledged. First, the review is based entirely on case reports and case series, which inherently carry a high risk of selection and publication bias. Most reports lacked standardized follow-up and did not consistently report clinical metrics or treatment protocols, reducing comparability across cases. The heterogeneity in clinical presentations, diagnostic workups, and treatment modalities further precluded a meta-analysis.

Additionally, a large number of potentially relevant articles could not be retrieved, possibly excluding additional informative cases. Finally, the JBI-based risk of bias assessment revealed that most reports lacked comprehensive data on adverse effects and long-term outcomes, further limiting the strength of evidence.

## 6. Conclusions

Salivary gland sarcoidosis predominantly affects middle-aged women, typically presenting as a painless parotid swelling and often serving as the initial sign of systemic disease. Diagnosis requires histopathological confirmation via biopsy, as serum ACE levels are insufficient alone. The prognosis is excellent. While systemic corticosteroids are the mainstay of treatment for significant or symptomatic disease, our findings strongly indicate that a conservative, “watchful waiting” approach is a viable and often underutilized strategy in select patients with mild, isolated gland involvement, given the notable rate of spontaneous remission. This condition must be considered in differential diagnoses for persistent salivary gland swellings to ensure accurate diagnosis, prevent unnecessary interventions, and tailor management to the patient’s specific clinical scenario.

## Figures and Tables

**Table 1:** PECOS inclusion and exclusion criteria.

Parameter	Inclusion	Exclusion
Population	Patients diagnosed with sarcoidosis	Patients diagnosed with other salivary gland lesions
Exposure	Salivary gland sarcoidosis cases	Sarcoidosis in other parts of the body
Comparison	N/A	N/A
Outcome	Clinical features Diagnostic methods Treatment and outcome	Incomplete demographic, histopathological, and clinical details.
Study design	Case reports and case series Full-text availability English language only	Review articles, editorials, animal studies and conference abstracts Non-English language articles Duplicates that may introduce bias

**Table 2:** Study characteristics of included studies.

Author/Year	Country	Age	Sex	Initial Symptoms	Duration (Months)	Location	ACE * Levels	Diagnosis	Treatment	Follow-Up and Outcome
Hoggins et al., 1969 [16]	UK	45	F	Firm and fleshy swelling involving left cheek and maxilla	12	Parotid	N/A	New	Surgical intervention + radiotherapy	2 years Resolved
Som et al., 1981 [17]	US	25	F	Left parotid enlargement	N/A	Parotid	N/A	Pre-existing	Surgical intervention	N/A
		56	M	Right parotid enlargement with facial palsy	N/A	Parotid	N/A	Pre-existing	Steroids	Resolved
		35	F	Left parotid enlargement	N/A	Parotid	N/A	Pre-existing	Steroids	Resolved
		55	F	Right parotid swelling	144	Parotid	N/A	New	Steroids	Resolved
Melsom et al., 1988 [18]	UK	57	F	Bilateral parotid swelling, dry mouth Difficulty chewing, swallowing, and speaking Malaise and tiredness, loss of weight	9	Parotid	Elevated	New	Steroids	Resolved
Ohtsuka et al., 2001 [19]	Japan	63	M	Painless and hard bilateral parotid and submaxillary node	132	Parotid	Decreased	New	Antibiotic	3 months Resolved
Surattanont et al., 2002 [20]	US	13	F	Painless, firm symmetrical bilateral parotid swelling	4	Parotid Labial salivary glands	Elevated	New	Steroids	N/A
Folwaczny et al., 2002 [12]	Germany	28	F	Bilateral firm painless enlargement of parotid glands, dry mouth, and difficulty chewing	0.5 (2 weeks)	Parotid	Elevated	New	None	N/A
Sinha and Gaur, 2004 [21]	India	49	F	Bilateral swelling at angles of the jaw, decreased salivation and loss of taste Low-grade fever, malaise and dry eyes	0.3 (10 days)	Parotid	Elevated	New	Non-surgical intervention + steroids	4 months Resolved
Vairaktaris et al., 2005 [22]	Greece	72	F	Xerostomia Painless parotid slow-growing enlargement unilateral	2	Parotid	N/A	New	Surgical intervention + steroids	15 months Resolved
		68	F	Enlarging lesion on left submandibular area, firm and immobile	3	Submandibular	N/A	New	Resection of mass + gland	12 months Resolved
		66	F	Painless submandibular swelling, gradual increase in size	2	Submandibular	N/A	New	Steroids	Reduction in swelling
Fatahzadeh and Rinaggio, 2006 [13]	USA	47	F	Severe xerostomia, burning tongue, and dysgeusia, dysphagia. Bilateral swelling.	9	Parotid Submandibular Minor salivary glands	Elevated	New	None	7 months Spontaneous remission
Yates and Dickenson, 2006 [23]	UK	39	F	Palpable lesion in anterior lobe of right parotid gland	1	Parotid	Elevated	New	Steroids	Resolved
McCormick et al., 2006 [24]	USA	51	F	Right-sided neck mass posterior to the angle of the mandible, with discomfort Gradual increase in size	1.5	Parotid	N/A	New	Surgical intervention	Resolved
Rudralingam et al., 2007 [25]	UK	50	M	Dry mouth and dry eyes Bilateral parotid gland swelling	12	Parotid	Elevated	New	Steroids	1 month Resolved
Poate et al., 2008 [26]	UK	27	M	Bilateral non-tender swelling of parotid glands, dryness of the mouth, fever, weight loss	3	Parotid	Elevated	New	Steroids	3 months Resolved
		35	F	Dry mouth and gritty eyes, fatigue	24	Labial minor salivary glands	Elevated	New	Steroids	1 year Resolved
Teymoortash et al., 2009 [27]	Germany	Avg: 35 Rng: 30–46	5 M 1 F	5 Bilateral enlargements 1 unilateral swelling 4 painful Firm, immobile mass	0.7–1.4 (3–6 weeks)	Parotid	Elevated	4 Pre-existing 2 New	Steroids	Resolved
Ahmad et al., 2010 [14]	Malaysia	29	M	Bilateral parotid enlargement, multiple cervical lymphadenopathies, pain while swallowing, malaise, low grade fever, night sweats and dry cough	3	Parotid	Normal	New	Steroids	6 months Resolved
Geraldes Filho 2010 [28]	Brazil	41	M	Painless, firm bilateral enlargement of the parotid and submandibular gland	months	Submandibular and parotid	N/A	New	None	Spontaneous remission
Banks et al., 2013 [29]	USA	13	M	Painless bilateral parotid enlargement and mild bilateral, anterior cervical adenopathy	1.2 (5 weeks)	Parotid	Normal	New	None	Spontaneous remission
Lowe et al., 2015 [30]	UK	22	F	Bilateral panuveitis and parotid enlargement Weight loss and fatigue	3	Parotid	N/A	New	Steroids	Resolved
Sharma et al., 2015 [31]	India	54	F	Swelling bilaterally, dryness of throat and eyes, and dry cough Decreased appetite and weight loss	6	Parotid	Elevated	New	Steroids	6 months Resolved
Chappity et al., 2015 [32]	India	52	F	Bilateral facial palsy, swelling in left parotid region, fever	24	Parotid	Elevated	New	Steroids	2 months Resolved
Lee et al., 2016 [33]	Korea	49	F	Right infra-auricular swelling	0.7 (3 weeks)	Parotid	N/A	New	Surgical intervention	Resolved
Broggi et al., 2017 [34]	Italy	60	F	Fever, complete facial palsy, nonpainful enlargement of left parotid. Deviation of tongue and mouth to the left	0.2 (1 week)	Parotid	Normal	New	Steroids	Resolved
Assiri and Al-Ahmari, 2018 [35]	Saudi Arabia	44	F	Painless right parotid swelling	4	Parotid	N/A	New	Surgical intervention	1 year Resolved
Brown et al., 2018 [36]	USA	5	M	Firm, painful bilateral parotid swelling	0.2 (7 days)	Parotid	Normal	New	Steroids	Resolved
Diamantopoulos et al., 2019 [37]	Greece	28	F	Painless hard parotid enlargement Facial palsy Malaise, low grade fever	0.5 (15 days)	Parotid	Normal	New	None	2 years Spontaneous remission
Derbel et al., 2021 [38]	Tunisia	52	F	Parotid enlargement, fever Dry mouth, dysphagia and dyspnea	24	Parotid	Elevated	New	Steroids	Recurred 7 years
Abraham et al., 2023 [39]	India	40	F	Bilateral parotid swelling, dry eyes and mouth	24	Parotid	Elevated	New	Steroids	9 months Resolved
Juras et al., 2024 [40]	Croatia	48	F	Painless firm parotid swelling Right swelling sublingual and neck area xerostomia	5	Parotid Sublingual	Elevated	New	Steroids	1 year Resolved
Total	12 countries	Range: 5–72 Mean (SD): 42.7 (16.4)	26 F 13 M	Swelling/enlargement: 32 Xerostomia: 13 Painless: 17 Fever: 7 Fatigue: 6 Pain: 6 Facial palsy: 4	0.2–144	Parotid: 32 Multiple: 4 Submandibular: 2 Minor salivary glands: 1	Elevated: 15 Normal: 5 Decrease: 1	New: 32 Pre-existing: 7	Non-surgical: 27 Surgical: 5 None: 5 Combination: 2	Resolved: 31 Spontaneous remission: 4 Unclear: 3 Recurred: 1

* angiotensin-converting enzyme. N/A—not available.

**Table 3:** JBI Critical Appraisal for quality assessment.

Author/Year	Demographic	History	Current Clinical Condition	Diagnostic Tests	Treatment Described	Post-Intervention Clinical Condition	Adverse Events	Takeaway Lessons	Overall Appraisal:
Hoggins et al., 1969 [16]	X		X	X	X	X		X	Moderate
Som et al., 1981 [17]	X		X	X	X			X	Moderate
Melsom et al., 1988 [18]	X		X	X	X	X		X	Moderate
Ohtsuka et al., 2001 [19]	X	X	X	X	X	X		X	High
Surattanont et al., 2002 [20]	X		X	X	X	X		X	Moderate
Folwaczny et al., 2002 [12]	X	X	X	X	X	X		X	High
Sinha and Gaur, 2004 [21]	X	X	X	X	X	X		X	High
Vairaktaris et al., 2005 [22]	X	X	X	X	X	X		X	High
Fatahzadeh and Rinaggio, 2006 [13]	X	X	X	X				X	Moderate
Yates and Dickenson, 2006 [23]	X	X	X	X	X	X		X	High
McCormick et al., 2006 [24]	X	X	X	X	X			X	High
Rudralingam et al., 2007 [25]	X	X	X	X	X	X		X	High
Poate et al., 2008 [26]	X	X	X	X	X			X	Moderate
Teymoortash et al., 2009 [27]	X		X	X	X	X		X	Moderate
Ahmad et al., 2010 [14]	X		X	X	X	X		X	Moderate
Geraldes Filho, 2010 [28]	X		X	X	X	X		X	Moderate
Banks et al., 2013 [29]	X		X	X	X	X		X	Moderate
Lowe et al., 2015 [30]	X		X	X	X	X		X	Moderate
Sharma et al., 2015 [31]	X		X	X	X	X		X	Moderate
Chappity et al., 2015 [32]	X		X	X	X	X		X	Moderate
Lee et al., 2016 [33]	X	X	X	X	X	X		X	High
Broggi et al., 2017 [34]	X		X	X	X	X		X	Moderate
Assiri and Al-Ahmari, 2018 [35]	X	X	X	X	X	X		X	High
Brown et al., 2018 [36]	X	X	X	X	X	X		X	High
Diamantopoulos et al., 2019 [37]	X		X	X	X	X		X	Moderate
Derbel et al., 2021 [38]	X		X	X	X	X	X	X	High
Abraham et al., 2023 [39]	X		X	X	X	X		X	Moderate
Juras et al., 2024 [40]	X		X	X	X	X		X	Moderate

**Table 4:** Differential Diagnosis of Salivary Gland Sarcoidosis—Key Comparison with Sjögren’s Syndrome and IgG4-Related Disease.

Feature	Salivary Gland Sarcoidosis	Sjögren’s Syndrome (SS)	IgG4-Related Disease (IgG4-RD)
Key Histopathology	Non-caseating granulomas (epithelioid histiocytes, giant cells) [47,48,49].	Focal lymphocytic sialadenitis (foci per 4 mm^2^) [54].	Storiform fibrosis, obliterative phlebitis, dense lymphoplasmacytic infiltrate with >10 IgG4+ plasma cells/HPF and >40% IgG4+/IgG+ ratio [54].
Serologic Markers	Serum ACE (elevated in ~41–78% of systemic cases; (limited sensitivity) [52,53].	Anti-SSA/Ro and anti-SSB/La antibodies (highly specific) [54].	Elevated serum IgG4 (in ~60–70% of cases) [54].
Glandular Symptoms	Painless or painful swelling; xerostomia common.	Sicca symptoms (dry mouth, dry eyes); glandular swelling.	Firm, painless glandular enlargement (e.g., submandibular glands often affected) [54].
Characteristic Extraglandular Manifestations	Heerfordt’s syndrome (uveitis, parotitis, fever, facial palsy) [34,36], pulmonary involvement, Löfgren’s syndrome [1,9].	Arthralgia/arthritis, Raynaud’s phenomenon, fatigue, cutaneous vasculitis [54].	Autoimmune pancreatitis, retroperitoneal fibrosis, sclerosing cholangitis, Riedel’s thyroiditis [54].
Typical Course and Prognosis	Often self-limiting; excellent response to corticosteroids [1,8,11].	Chronic, progressive autoimmune disorder [54].	Chronic, fibro-inflammatory; good response to corticosteroids but relapses common [54].
Diagnostic Approach	Biopsy is gold standard. Imaging (e.g., CXR/CT for systemic involvement) [11,46].	ACR/EULAR classification criteria (serology, ocular/staining, histology) [54].	Comprehensive diagnostic criteria (histology, serology, imaging of characteristic organ involvement) [54].

## Data Availability

No new data were created or analyzed in this study.
